# Editorial: Genomic Instability and Neurodegeneration

**DOI:** 10.3389/fnagi.2022.940459

**Published:** 2022-06-08

**Authors:** Julia Fuchs, Bjoern Schwer, Sherif F. El-Khamisy

**Affiliations:** ^1^Center for Interdisciplinary Research in Biology (CIRB), CNRS, INSERM, Collège de France, Université PSL, Paris, France; ^2^Eli and Edythe Broad Center of Regeneration Medicine and Stem Cell Research, University of California, San Francisco, San Francisco, CA, United States; ^3^Bakar Aging Research Institute, University of California, San Francisco, San Francisco, CA, United States; ^4^Kavli Institute for Fundamental Neuroscience, Weill Institute for Neurosciences, University of California, San Francisco, San Francisco, CA, United States; ^5^Department of Neurological Surgery, University of California, San Francisco, San Francisco, CA, United States; ^6^Healthy Lifespan Institute and the Institute of Neuroscience, School of Biosciences, University of Sheffield, Sheffield, United Kingdom; ^7^Institute of Cancer Therapeutics, University of Bradford, Bradford, United Kingdom

**Keywords:** neurodegeneration, DNA damage, genomic instability, DNA repair, aging

Genomic instability can be defined as an increased probability in accumulating genome damage, acquired either through a defect in the repair of such damage, or an accumulation of inductive triggers. While genomic instability is a well-established hallmark of cancer and aging, its relevance for neurodegeneration remains less well understood. This is about to change as we begin to recognize that age is intrinsically linked to the most frequent neurodegenerative diseases, and that neurons due to their particular metabolism and their non-dividing state, are particularly prone to accumulate DNA damage. It is thus crucial to investigate the origins of genomic instability in neurons, how this instability might trigger neurological diseases and to identify possible targets for intervention.

In their review entitled “*SIRT6 Through the Brain Evolution, Development, and Aging*,” Garcia-Venzor and Toiber discuss the hypothesis that the deacetylase Sirt6 acquired additional functions throughout evolution to counteract an increasing burden of genomic instability due to higher metabolic and proliferative brain activities linked to a complexified brain structure and function. To this end, Sirt6 operates at multiple levels including the post-translational modification of histone proteins and the silencing of transposable elements. The association of the latter with genomic instability and neurodegeneration is reviewed in “*Retrotransposons as a source of DNA damage in neurodegeneration*” by Peze-Heidsieck et al. The authors suggest that genomic instability induced by LINE-1 retrotransposon activation in neurons could molecularly link aging and neurodegeneration via aging-induced heterochromatin disorganization, subsequent de-repression of transposable elements and LINE-1-related genomic instability leading to neurodegeneration.

Topoisomerase-generated genomic instability, another endogenous source of DNA damage, is discussed in the review “*Topoisomerase-mediated DNA damage in neurological disorders*” by Crewe and Madabhushi. Here, the authors highlight the sources of such damage, the repair pathways involved and the importance of these processes for the pathogenesis of neurological disorders. Notably, congenital defects in DNA repair can affect the immune system, the skin or predispose to cancer, but a perturbation of the nervous system is common to all genetic DNA repair deficiency syndromes suggesting a particular vulnerability of brain cells to dysfunctional DNA repair.

While accumulating evidence suggests that neurodegenerative diseases are associated with genomic instability and a deficiency in DNA repair, congenital syndromes often affect the cerebellum and present with progressive ataxia. The origins of this selective vulnerability of the cerebellum remains an open unanswered question. The role of a specific DNA damage response pathway in the survival and function of Purkinje cells, the largest cerebellar neurons whose dysfunction or degeneration causes ataxia, is investigated in the original research article “*The essential DNA damage Response Complex MRN is dispensable for the survival and function of Purkinje neurons*” by Ding et al. While mutations in MRN complex repair proteins cause ataxic phenotypes in humans and in mice when deleted at the neuronal progenitor stage, the authors provide genetic evidence that the two MRN complex components Nbs1 and Mre11 play non-essential functions in post-mitotic Purkinje neurons, despite the fact that their absence does elicit DNA damage response impairments. As these components are highly expressed in Purkinje neurons, the authors speculate on reasons for the absence of a neurodegenerative or behavioral phenotype and suggest non-canonical functions of these highly expressed proteins which remain to be explored.

Post-mitotic neurons likely accumulate DNA damage over their lifetime and with age, the efficiency of DNA repair pathways starts to decline. In “*DNA damage, defective DNA repair and neurodegeneration in Amyotrophic Lateral Sclerosis*,” Konopka and Atkin review recent evidence linking DNA damage and defective DNA repair to the pathogenesis of this age-related neurodegenerative disease that affects upper and lower motor neurons and which very recently has been genetically linked to proteins related to DNA repair.

Finally, in an opinion article, Iourov and Vorsanova speculate on an impact of SARS-CoV-2 on genomic instability in the brain. Based on emerging evidence that the SARS-CoV-2 virus affects brain function, they hypothesize that virus-related genomic instability might increase the risk for the development of late-onset neurodegenerative diseases.

Thus, a picture emerges in which external sources like viruses along with multiple internal sources can trigger DNA damage in the brain. These internal sources include dysregulation of epigenome guards and expression regulators like Sirt6 with aging, the dependence on the proper functioning of DNA repair proteins at defined developmental stages, transposable retroelement activation with aging, and the dysregulation of the repair of topoisomerase activity-related DNA damage associated with transcription. Together, an imbalance between the occurrence of DNA damage and the efficiency of DNA repair processes is emerging as a potential driving force in the pathogenesis of several neurological diseases including age-related neurodegenerative diseases. Consequently, preventive approaches that limit external or internal DNA damage sources might prove to be beneficial for brain health. Indeed, in the future, interventions designed to promote DNA repair might be developed based on the increasing knowledge of DNA damage and repair in brain cells. However, certain types of DNA damage may have physiological functions, either during transcriptional regulation or *via* formation of genetic mosaicism by DNA rearrangements or the insertion of transposable elements in neuroprogenitors, which could be beneficial to create neuronal diversity and increase brain plasticity. Paradoxically, the brain appears to be particularly dependent on an extremely well equilibrated balance between damage and repair but does not contain the full repertoire of DNA repair pathways. Elucidating the interplay between brain function, aging, and DNA damage repair will require significant effort but is likely to be impactful for preventing neurological and neurodegenerative disorders in humans in the future.

## Author Contributions

All authors listed have made a substantial, direct, and intellectual contribution to the work and approved it for publication.

## Funding

JF is supported by grants from the Fondation de France (00086320), Fondation du Collège de France, the National French Agency for Research (Agence Nationale de la Recherche; ANR-20-CE16-0022 NEURAGE), the Fondation pour la Recherche sur Alzheimer's" (FRA) and the Fondation NRJ. BS is supported by the National Institute On Aging of the National Institutes of Health under award numbers R01AG064363 and R56AG071857, the UCSF Program for Breakthrough Biomedical Research (which is partially funded by the Sandler Foundation), a Kimmel Scholar Award of The Sidney Kimmel Foundation, a Carol and Gene Ludwig Award for Early Career Research, a Bakar Aging Research Institute Investigator Award and holds the Suzanne Marie Haderle and Robert Vincent Haderle Endowed Chair at UCSF. Work in El-Khamisy lab is supported by a Wellcome Trust Investigator Award (103844) and a Lister Institute of Preventative Medicine Fellowship (137661).

## Conflict of Interest

The authors declare that the research was conducted in the absence of any commercial or financial relationships that could be construed as a potential conflict of interest.

## Publisher's Note

All claims expressed in this article are solely those of the authors and do not necessarily represent those of their affiliated organizations, or those of the publisher, the editors and the reviewers. Any product that may be evaluated in this article, or claim that may be made by its manufacturer, is not guaranteed or endorsed by the publisher.

